# Size-dependent interactions between calciprotein particles and vascular endothelium

**DOI:** 10.1016/j.mtbio.2025.101599

**Published:** 2025-02-19

**Authors:** Zeping Zhang, Xinyue Wang, Caihao Huang, Meixia Wang, Wei Cui, Liang Hao, Rui Yang, Hong-hui Wang, Xing Zhang

**Affiliations:** a Institute of Metal Research, Chinese Academy of Sciences, Shenyang, Liaoning 110016, China; b School of Materials Science and Engineering, University of Science and Technology of China, Hefei, Anhui 230026, China; c School of Materials Science and Engineering, Dalian University of Technology, Dalian, Liaoning 116024, China; d State Key Laboratory of Chemo/Bio-Sensing and Chemometrics, College of Biology, Hunan University, Changsha, Hunan 410082, China; e School of Forensic Medicine, China Medical University, Shenyang, Liaoning 110026, China

**Keywords:** Endothelium, Calciprotein nanoparticles, Chronic kidney disease, Biomimetic synthesis

## Abstract

The underlying mechanisms governing the interactions between nanoparticles and vascular endothelial barrier remain largely unexplored, which is crucial for the optimal design of nanoparticles for clinical applications. In this study, the size-dependent interactions between calciprotein particles (CPPs) and endothelial cells (ECs) were investigated using a rat model of chronic kidney disease (CKD) induced by 5/6 nephrectomy. Two primary types of CPP1 were studied: small-sized CPP1 (S-CPP1, <50 nm) and larger CPP1 (L-CPP1, <100 nm), detected three and five weeks post-surgery, respectively. By adjusting the amounts of Ca^2+^, HPO_4_^2−^ and H_2_PO_4_^−^ ions in Dulbecco's Modified Eagle Medium supplemented with 10 % (V/V) fetal bovine serum and 1 % (V/V) Pen-Strep solution, S-CPP1 (<50 nm) with an elliptical shape, L-CPP1 (50–100 nm), and secondary CPPs (CPP2, >100 nm) with a needle-like crystalline structure, resembling endogenous CPPs, were synthesized. The results showed that S-CPP1 significantly increased endothelial permeability at concentrations of 445 μg/mL and 890 μg/mL, thereby disrupting the integrity of the endothelial barrier formed by a confluent monolayer of ECs. Immunofluorescence analysis revealed that L-CPP1 was internalized by ECs via endocytosis, while S-CPP1 disrupted VE-cadherin junctions, leading to irregular cell morphology and widened intercellular gaps. These structural changes likely contribute to medial calcification as CPPs accumulate within the circulatory system. In conclusion, the findings underscore that the interaction between CPPs and the vascular endothelium is strongly size-dependent, with significant implications for vascular system health and the design of nanoparticle-based therapies.

## Graphical abstract

**Figure:**
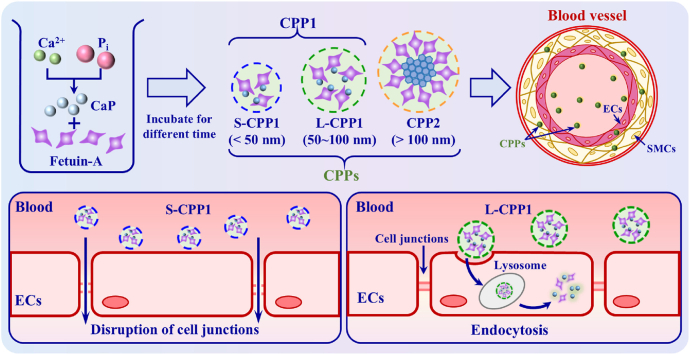

## 1 Introduction

Nanoparticles have been extensively developed in the biomedical field [[1], [2], [3]], with a wide range of applications such as tumor treatment [4,5], medical imaging [6,7] and wound dressings [8]. The administration routes for nanoparticles typically include intravenous, oral and transdermal methods [9]. Once injected into the human body, most nanoparticles will eventually enter the blood circulation [10]. It is well established that the vascular endothelium is a dynamic monolayer lining the innermost part of the vascular system, serving as the primary barrier to nanoparticle-based drug delivery within the circulatory system [3,9,11]. However, the interactions between nanoparticles and endothelial cells (ECs) in the vascular endothelium remain controversial.

The physical and chemical properties of nanoparticles are known to significantly affect their interactions with ECs [9,12,13]. For example, Carlson et al. demonstrated that smaller silver nanoparticles (∼15 nm) can elicit more reactive oxygen species, thereby causing higher cytotoxicity compared to larger particles (∼30–55 nm) [14]. Similarly, Setyawati et al. proved that titanium dioxide nanoparticles [5,15] and gold nanoparticles [16] can disrupt VE-cadherin junctions between ECs, leading to endothelial leakage. However, current studies on the interactions between nanoparticles and ECs typically rely on artificial *in vitro* models, such as transwell models [5,16] and microfluidic models [17,18]. While these approaches provide valuable insights, they oversimplify the complexity of *in vivo* microenvironments, failing to capture the dynamic and multifaceted nature of the vascular system.

To address this limitation, naturally occurring endogenous calciprotein particles (CPPs), which are directly implicated in vascular calcification, particularly in chronic kidney disease (CKD) patients [19,20], are used to investigate the interactions between CPPs and ECs. CPPs form naturally in the bloodstream through the aggregation of calcium phosphate and proteins such as fetuin-A. These aggregates develop into primary CPPs (CPP1, <100 nm), which contain amorphous calcium phosphate. Subsequently, these primary CPPs undergo a transition into secondary CPPs (CPP2, >100 nm), characterized by the presence of crystalline hydroxyapatite (HAP) [[21], [22], [23], [24]]. Previous findings have demonstrated a strong correlation between the concentration of CPPs in the blood and the extent of medial calcification in blood vessels [23,25,26]. However, the mechanisms through which CPPs cross the endothelial barrier and contribute to intimal calcification remain unclear, particularly regarding the effects of particle sizes.

In this study, endogenous CPPs were characterized utilizing a 5/6 nephrectomy rat model. Large quantities of different CPPs were synthesized to replicate various sizes of endogenous CPPs. Both *ex vivo* blood vessel model and *in vitro* transwell models were employed to investigate the size-dependent interactions between CPPs and ECs. The findings elucidate the importance of interactions between naturally occurring nanoparticles and ECs, and underscore how the nanoparticle size influences their ability to traverse the vascular endothelium.

## 2 Experimental details

### 2.1 Construction of the 5/6 nephrectomy rat CKD model

All experiments were performed in accordance with the “Regulations on the Administration of Experimental Animals by the Ministry of Science and Technology of the People's Republic of China.” All procedures involving animal experiments were reviewed and approved by the Hunan University Committee on Experimental Animal Ethics and the Hunan Provincial Center for Experimental Animals Animal Experiment Ethics Committee. Male Sprague-Dawley (SD) rats, aged 8–10 weeks, were housed in a temperature-controlled environment (22 ± 2 °C) with a 12-h light/dark cycle. The rats were provided with ad libitum access to standard rodent chow and water. All animals were monitored daily to ensure their health and well-being, and efforts were made to minimize discomfort throughout the study.

A rat model of CKD was established by 5/6 nephrectomy using six 8-week-old male SD rats. The rats were anesthetized using isoflurane, and two-thirds of the right kidney's upper and lower poles were first cut off. One week later, the left kidneys were removed and sutured with 3 mm stitches, marking the successful establishment of the 5/6 nephrectomy rat model. Approximately 2 mL of blood was drawn from the tail vein of each rat post-surgery for three weeks. The supernatant of the blood was then separated by centrifugation at 3000*g* for 15 min, followed by high-speed centrifugation (High-speed centrifuge, HC-3018, Anhui USTC Zonkia Scientific Instruments Co., Ltd., Hefei, China) at 100,000 g at 4 °C for 2 h to precipitate CPPs. Blood samples containing CPPs were collected from the inferior vena cava of rats post-surgery for five and six weeks. Post-collection, the blood vessels were flushed with chilled D-PBS, and the abdominal aortas were excised, immediately immersed in 2.5 % (W/V) glutaraldehyde (GA) solutions, and stored at 4 °C.

### 2.2 Biomimetic synthesis of CPPs

Prior to preparation of small-size CPP1 (hereafter named S-CPP1), stock solutions of 0.38 mol/L CaCl_2_, 0.142 mol/L Na_2_HPO_4_, and 0.272 mol/L NaH_2_PO_4_·2H_2_O were prepared. The solutions were sterilized using a 0.22 μm filter and stored at 4 °C. 0.5 mL CaCl_2_ solution, 0.37 mL Na_2_HPO_4_ solution, and 0.33 mL NaH_2_PO_4_·2H_2_O solution were then sequentially added into 50 mL Dulbecco's Modified Eagle Medium (DMEM, Gibco, Pittsburgh, U.S.A.) supplemented with 10 % (V/V) fetal bovine serum (FBS, Gibco, Pittsburgh, U.S.A.), and 1 % (V/V) Pen-Strep solution (Genview Scientific, Inc., Calimesa, U.S.A.) (DMEM complete medium). The mixed solution was then reacted for 4 h, followed by sterilization using a 0.22 μm filter membrane to obtain a solution of S-CPP1.

Before synthesizing large-size CPP1 (hereafter named L-CPP1) and secondary CPPs (hereafter named CPP2), stock solutions of 0.2 M CaCl_2_, 0.142 M Na_2_HPO_4_, and 0.272 M NaH_2_PO_4_·2H_2_O were prepared. These solutions were sterilized using a 0.22 μm filter and stored at 4 °C. 0.5 mL CaCl_2_ solution, 1.5 mL Na_2_HPO_4_ solution, and 0.5 mL NaH_2_PO_4_·2H_2_O solution were then sequentially added into 100 mL of DMEM complete medium. The mixture was incubated in a thermostatic shaking incubator (ZDP-150, 110 rpm, Jinghong Experimental Equipment Co., Ltd., Shanghai, China) at 37 °C, 110 rpm for 2 h to obtain L-CPP1 and for 14 days to obtain CPP2. The suspension was centrifuged at 12,000 g for 30 min to collect CPPs.

All chemical reagents were obtained from Sinopharm Chemical Reagent Co., Ltd. (Beijing, China).

### 2.3 Construction of *ex vivo* blood vessel model

For the construction of *an ex vivo blood vessel model*, the rats, aged 8–10 weeks, were euthanized, and their abdominal aortas were carefully excised. The vessels were quickly placed in a culture box containing DMEM with 10 % (V/V) FBS and 2 % (V/V) Pen-Strep solution. Each vessel was connected to the adapters within the culture box and secured with hemostatic clips. A circulation path was created outside the adapters utilizing a catheter. The system was subjected to a continuous flow of DMEM complete medium supplemented with 10 μg/mL CPPs at 22 mL/min controlled by a peristaltic pump. The experimental setup was placed in an incubator (HREAcell 150i, Thermo Fisher Scientific Inc., Waltham, U.S.A.) to simulate physiological conditions. The medium containing CPPs was refreshed every day. After eight days of circulation, the vessels were harvested and fixed in 2.5 % (W/V) GA solutions. The harvested tissues were stained with hematoxylin-eosin (H&E).

### 2.4 Morphology of the vessels and endogenous CPPs

The GA-fixed abdominal aortas from the rat model of 5/6 nephrectomy were dehydrated in graded acetone concentrations (50 %, 70 %, 90 %, and 100 %) at 4 °C, for 20 min each. The vessels were subsequently immersed in acetone and epoxy resin mixtures at 3:1 and then 1:3 vol ratios, each for 30 min, before embedding in pure resin for 2 h. These resin-embedded vessels were cut into blocks (∼2 mm^3^), placed into molds, and cured at 80 °C for 12 h. Subsequently, ultrathin sections (70 nm) were prepared with an ultramicrotome (Leica EM UC7, Leica, Wetzlar, Germany). These sections were then transferred on a TEM microgrid support film and dried for further TEM analysis (Tecnai G2 F20, FEI, Hillsboro, U.S.A.).

The morphology of endogenous CPPs was observed using a field emission scanning electron microscope (SEM, Inspect F50, FEI, Hillsboro, U.S.A.) at 20 kV, and elemental compositions were determined by the energy dispersive X-ray spectroscopy (EDS, Oxford INCAX 7582, Oxford, U.K.). The lyophilized CPP powder was resuspended and sonicated in deionized water (100 Hz, 5 min), and a droplet was then placed on a carbon-coated copper mesh for drying and subsequent analysis by TEM.

For the *ex vivo blood vessel model*, GA-fixed vessels were dehydrated in alcohol with a series of concentrations (50 %, 70 %, 90 %, 95 %, and 100 %) at 4 °C for 20 min each. Subsequently, the vessels were longitudinally sectioned, and their endothelial layers were affixed, facing upwards, on a sample plate coated with conductive tape for SEM analysis.

### 2.5 Characterization of synthetic CPPs

The crystal phases of synthetic CPPs were determined by X-ray diffraction (XRD, SmartLab, Rigaku, Tokyo, Japan) with monochromatic Cu Kα1 radiation in the range of 20°–70° with a step size of 0.02°. Fourier transform infrared spectroscopy (FTIR, TENSOR 27, Bruker, Karlsruher, Germany) was used to analyze the chemical bonding information of CPPs, and the correlation characterization was carried out in the wavenumber range of 400–4000 cm^−1^. The calcium and phosphorus contents in the CPPs suspension were determined using inductively coupled plasma-optical emission spectrometry (ICP-MS, Prodigy, Leeman Labs, Hudson, U.S.A.). The CPPs were completely dissolved by mixing with 5 wt% hydrochloric acid solution and each sample was tested three times.

Thermogravimetric-Differential Thermal Analysis (TG-DTA) was conducted with a STA 449F3 thermal analyzer (NETZSCH, Bavaria, Germany) to assess organic content in CPPs, heating from 30 °C to 900 °C at 10 °C/min under air flow. Particle size distribution and zeta potential were measured using a ZEN3690 particle size analyzer (Malvern, Worcestershire, UK).

### 2.6 Cell culture and viability assays

Human umbilical vein endothelial cells (HUVECs), obtained from Tongpai Biotechnology Co., Ltd., Shanghai, China, were cultured in Endothelial Cell Medium (ECM, ScienCell, San Diego, U.S.A.), supplemented with 10 % (V/V) FBS, 1 % (V/V) Endothelial Cell Growth Supplement (ECGS), and 1 % (V/V) Pen-Strep solution (ECM complete medium), which was replaced every 2 days. Prior to use in experiments, L-CPP1 was sterilized using UV irradiation. L-CPP1 at different concentrations (0, 800, 1600 μg/mL) were then evenly dispersed in ECM and co-cultured with HUVECs.

Cell proliferation was evaluated using the Cell Counting Kit-8 reagent (CCK-8, Genview Scientific, Inc., Calimesa, U.S.A.). HUVECs (3 × 10^3^ cells/well) were seeded into 96-well plates (n = 3). After culture for 24 h, the medium was replaced with L-CPP1 suspension at various concentrations and further incubated for 1, 3, and 5 days. At each time point, the CCK-8 reagent was added to the medium at a 1:10 volumetric ratio and incubated for 3 h before measuring absorbance at 450 nm with a microplate reader (Multiskan Go, Thermo Fisher Scientific, Waltham, U.S.A.).

### 2.7 Endocytosis of L-CPP1 by HUVECs

L-CPP1 was mixed with fluorescein isothiocyanate (FITC, Sigma-Aldrich, Shanghai, China). In a dark environment, 5 mg of FITC was added to 100 mL of DMEM complete medium, and the mixture was stirred for 30 min. The subsequent steps followed those described in Section 2.2 to obtain FITC-labeled L-CPP1 (FITC-L-CPP1). FITC-L-CPP1 was co-cultured with HUVECs, and the distribution of L-CPP1 at various time points was observed using a confocal laser microscope (CLSM) (Nikon A1R + Ti2-E, Nikon Instruments, Tokyo, Japan) to assess the interaction with cells.

500 μL of 1 % sterile gelatin solution (Leagene Biotechnology Co., Beijing, China) was added to the confocal dishes for 30 min, which was then removed to obtain the gelatin coating. HUVECs (1 × 10^4^ cells per dish) were cultured in these dishes with ECM medium supplemented with 10 % (V/V) FBS for 48 h and further cultured with FITC-L-CPP1 (200 μg/mL) in serum-free ECM for 0, 1, and 4 h. After removing the culture medium, 200 μL of 50 nM Lyso-Tracker Red (Beyotime Biotechnology, Shanghai, China) was added to the culture dish and incubated at 37 °C in darkness for an hour. 200 μL of 10 μg/mL Hoechst 33342 (Dingguo Changsheng Biotechnology, Beijing, China) was incubated with the cells at 37 °C for 10 min for nuclear staining. Each step was thoroughly washed with D-PBS (Genview Scientific, Inc., Calimesa, U.S.A.). Observations were subsequently performed using a CLSM (λ_(FITC)_: 495–525 nm; λ_(Lyso-Tracker Red)_: 577–590 nm; λ_(Hoechst 33342)_: 346–460 nm).

### 2.8 Permeability assay of HUVECs

The transwell system was employed to assess the permeability of HUVECs when cultured with L-CPP1/S-CPP1. HUVECs were seeded into the apical chamber (0.4 μm pore size, polyester membrane) at a density of 5 × 10^4^ cells/chamber and incubated for 48 h to form a dense layer. 550 μL of ECM complete medium was then added to the basolateral chamber, while 150 μL of different concentrations of L-CPP1 (0, 445, 890 μg/mL) or S-CPP1 in serum-free DMEM (0, 445, 890 μg/mL) was added to the apical chamber. After incubation for 1 and 4 h, the apical chambers were washed three times with D-PBS, which were transferred to a new 24-well plate with the basolateral chamber pre-filled with 550 μL D-PBS per well. 150 μL of 1 mg/ml D-PBS solution containing 70 kDA FITC-dextran (MKbio, Shanghai, China) was then added to each apical chamber in the dark. After incubation for 45 min, the fluorescence intensity of the medium in the apical chambers and basolateral chambers were measured using a full-wavelength microplate reader at excitation and emission wavelengths of 485 and 520 nm (Multiskan Go, Thermo Fisher Scientific, Waltham, U.S.A.). ECs permeability was calculated using the following equation (1):

(1) $$\text{Pd}=A_{b}/t\times 1/S\times v/A_{a}$$

where Pd is the permeability coefficient of dextran, t is the time in seconds, S refers to the membrane area of the chamber (cm^2^), A_a_ and A_b_ and represent the absorbance of the apical and basolateral chambers, respectively. v indicates the volume of the basolateral chamber.

### 2.9 Immunofluorescence staining of VE-cadherin in HUVECs

HUVECs (passages 4–6) were seeded at 7 × 10^4^ cells/well and cultured for 48 h. The ECM complete medium supplemented with S-CPP1 (0, 445 μg/mL) was then added to the wells and incubated for 4 h (n = 3). For VE-cadherin staining, the cells underwent fixation with 4 % paraformaldehyde (Dingguo Changsheng Biotechnology, Beijing, China) for 15 min, permeabilization with 0.1 % Triton X-100 (Dingguo Changsheng Biotechnology, Beijing, China) for 15 min, and blocking with goat serum for 20 min. The addition of VE-cadherin antibody (5 μg/mL, Annoron Biotechnology, Beijing, China) for an hour, biotinylated anti-rabbit IgG (1:100) for 30 min, and SABC-FITC (1:100) for another 30 min were performed. DAPI (100 ng/mL, Beyotime Biotechnology, Shanghai, China) was used for nuclei staining for 15 min. Each step was interspersed with three D-PBS washes.

### 2.10 Calcification of vascular smooth muscle cells (SMCs) induced by CPPs

Rat aortic thoracic smooth muscle cells (A7r5), obtained from the cell bank of Chinese Academy of Sciences in Shanghai, were seeded in a 24-well plate at a density of 2 × 10^4^ cells/well and cultured for 48 h. At about 80 % confluence, the culture medium was replaced with 1 mL DMEM complete medium containing 445 μg/mL S-CPP1, and incubated for 5, 7, and 9 days. A7r5 cells cultured with normal culture medium for 7 days were treated with 1 mL of DMEM complete medium supplemented with 200 μg/mL CPP2 for 1 day, serving as the positive control in this experiment. Cells were fixed with 4 % paraformaldehyde for 15 min and stained with 0.2 % Alizarin Red S (Beijing Solarbio Science & Technology, Beijing, China) for an hour. The stained nodules were then dissolved in 10 % hexadecyl pyridinium chloride (Sigma-Aldrich, St. Louis, U.S.A.), and the optical density (OD) was measured at 562 nm using the microplate reader.

### 2.11 Statistical analysis

All data were presented as the mean ± standard deviation from at least three experiments. The significant difference among multiple groups was analyzed with one-way ANOVA followed by Tukey's *post hoc* test. A value of *p* < 0.05 was considered to be statistically significant.

## 3 Results

### 3.1 Endogenous CPPs in the 5/6 nephrectomy rat model

The formation and progression of endogenous CPPs in the 5/6 nephrectomy rat model were tracked over time. appsec1Fig. S1 shows the endogenous CPPs isolated from the different duration rat CKD model induced by 5/6 nephrectomy. Ellipsoid-shaped CPPs with a particle size of less than 50 nm (S-CPP1) were detected in the bloodstream three weeks post-surgery. By five weeks post-surgery, CPPs with an approximate diameter of 75 nm (less than 100 nm) were observed, some of which exhibited hexagonal shapes (L-CPP1, red arrows). These findings suggest that early mineral metabolism disorders emerged within three weeks of nephrectomy, as indicated by the presence of amorphous S-CPP1. By the fifth week, L-CPP1 particles (50–100 nm) likely formed through the further aggregation of S-CPP1. Moreover, the development of hexagonal shapes indicated the onset of L-CPP1 partially crystallized. Over time, the mineral phase underwent a solid-solid phase transition, rearranging into needle-like HAP crystals during the formation of CPP2 particles exceeding 100 nm in size.

**Fig. 1:**
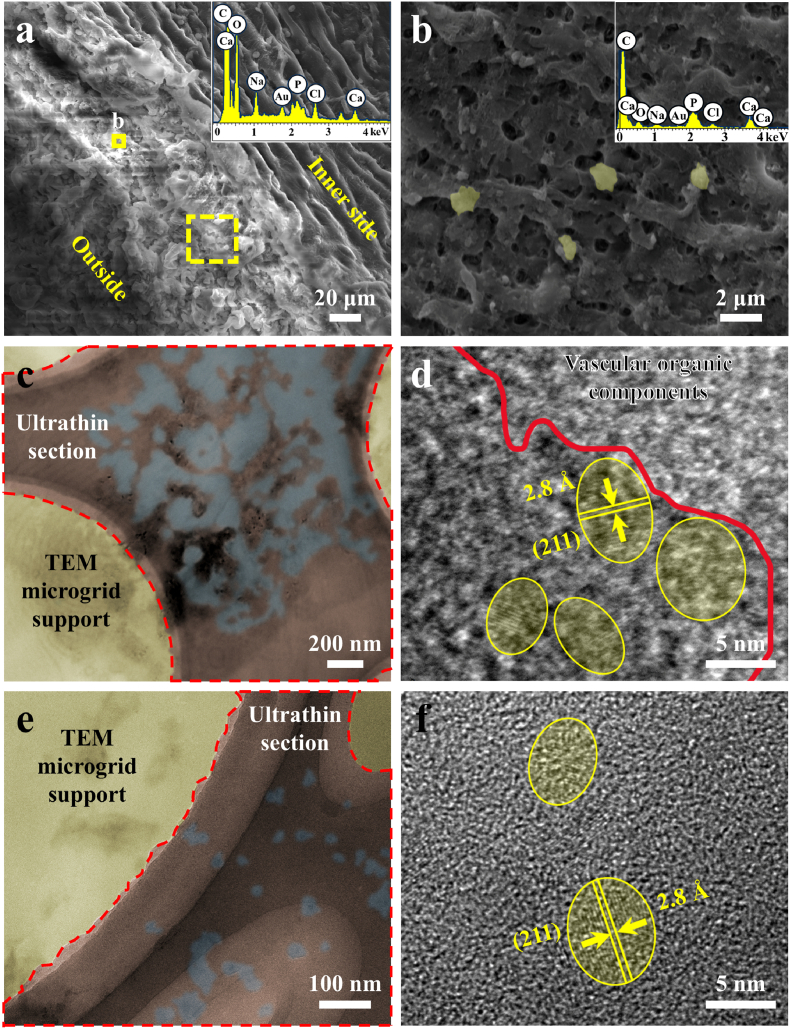
Microscopic analysis of the abdominal aorta at different time points following the establishment of the 5/6 nephrectomy rat model. (a, b) SEM image of the cross-section of the abdominal aorta wall five weeks post-surgery, with corresponding EDS spectra. (b) is a magnified view of the area outlined in yellow in (a). (c) TEM image of an ultrathin section of the abdominal aortic wall five weeks post-surgery, with a high-resolution image of calcified particles shown in (d). (e) TEM image of an ultrathin section of the abdominal aortic wall six weeks post-surgery, with a high-resolution image of calcification spots shown in (f). TEM microgrid support, ultrathin section of the abdominal aorta, and mineral components are highlighted in yellow, red, and blue, respectively, based on gray values in (c) and (e).

To evaluate the distribution of CPPs in the blood vessels, cross-sections of the rat abdominal aorta were examined five weeks post-surgery. As shown in Fig. 1a and b, particles with different contrasts compared to the surrounding vascular tissue were observed. EDS analysis confirmed that these particles contained calcium (Ca), phosphorus (P), and oxygen (O), indicating the presence of calcification sites.

To precisely characterize the calcified particles within vessel cross-sections, ultrathin sections of the abdominal aorta from the nephrectomy rats were examined using TEM. Fig. 1c presented an ultrathin section of the abdominal aorta (region by the red dotted line) attached to the microgrid support (yellow region), with some higher contrast areas in the blue region, indicating the mineral components. The high-resolution TEM image (Fig. 1d) showed that these regions contained multiple nanoparticles of 5–20 nm (yellow ellipsoids), with an interplanar spacing of 2.8 Å, corresponding with the (211) crystal plane of HAP (JCPDF# 09–0432), which indicates early calcification-like particles in the vessel cross-sections following the 5/6 nephrectomy for five weeks. After six weeks post-surgery (Fig. 1e), the mineral deposits embedded within the vascular organic matter were visible as clusters formed by the aggregation of numerous HAP nanoparticles, ranging from 5 to 20 nm in size (Fig. 1f).

In summary, the formation and progression of endogenous CPPs in the 5/6 nephrectomy rat CKD model were study. Three weeks post-surgery, ellipsoid-shaped S-CPP1 (<50 nm) were detected, indicating early disturbances in mineral metabolism in the blood. By the fifth week, L-CPP1 particles (∼50–100 nm) formed, with some particles exhibiting crystallization and showing a transition toward CPP2. At the same time, aggregation of 5–20 nm calcified particles was also observed in the rat aorta, with composition and size comparable to that of S-CPP1. These findings suggest that early signs of small calcified particle deposition in the vasculature can be detected following nephrectomy, likely linked to endogenous CPPs.

### 3.2 Microstructure and chemical composition of synthetic CPPs

While the endogenous CPPs extracted from the 5/6 nephrectomy rat model closely resemble those found in CKD patients, the quantity of CPPs directly obtained from rat blood samples was insufficient for comprehensive analysis. To address this limitation, synthetic CPPs were prepared for further study. The synthesis process was based on the calcium (Ca) and phosphorus (P) elemental content of endogenous CPPs in CKD models [20,27,28], following the method described in Section 2.2.

**Fig. 2:**
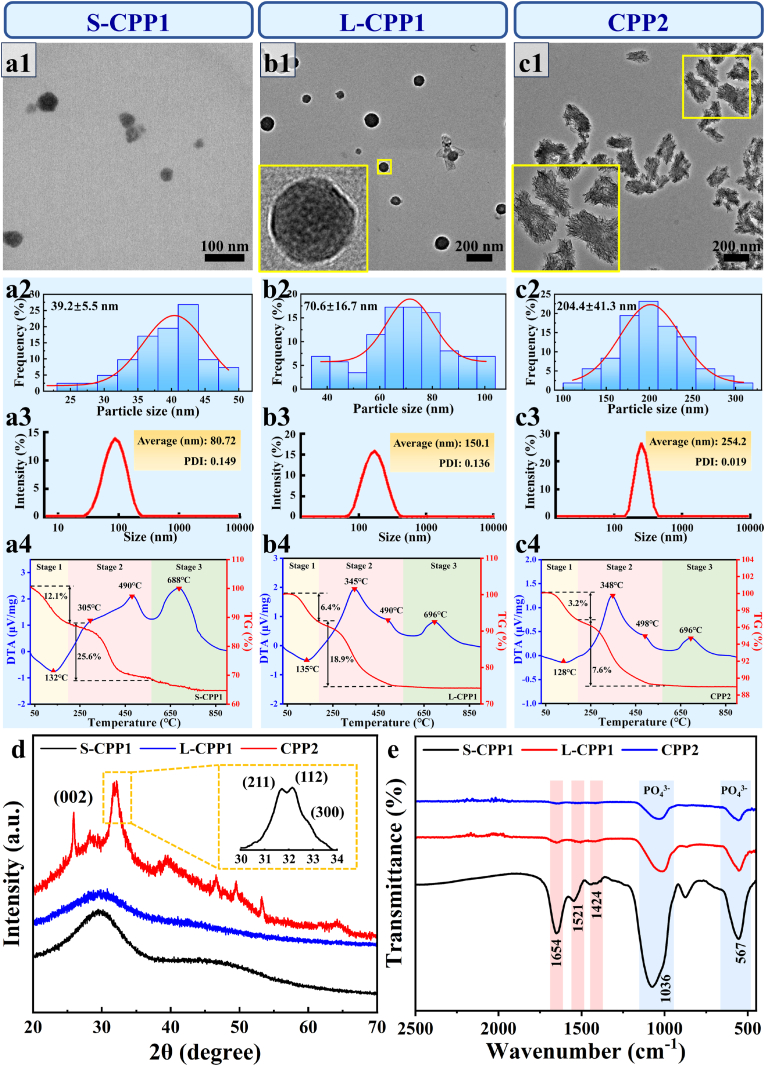
Microstructure and chemical composition of synthetic CPPs. S-CPP1: (a1) TEM bright-field images, (a2) particle size, (a3) hydration particle diameters and (a4) TG-DTA analysis. L-CPP1: (b1) TEM bright-field images, (b2) particle size, (b3) hydration particle diameters and (b4) TG-DTA analysis. CPP2: (c1) TEM bright-field images, (c2) particle size, (c3) hydration particle diameters and (c4) TG-DTA analysis. XRD patterns (d) and FTIR spectra (e) of synthetic S- CPP1, L-CPP1 and CPP2.

Fig. 2a1 shows the TEM bright-field image of synthesized S-CPP1 nanoparticles, revealing the ellipsoidal shape with a mean diameter of 39.2 ± 5.5 nm (Fig. 2a2), slightly smaller than the hydrated diameter measured by laser diffraction analysis (Fig. 2a3). EDS analysis (Fig. S2) confirmed that synthesized S-CPP1 contains Ca, P, and O elements, consistent with the composition of endogenous S-CPP1. TG-DTA analysis was conducted to assess the organic content of S-CPP1 (Fig. 2a4). The DTA curve (blue line) displayed an endothermic peak, followed by three exothermic peaks during the heating process from 30 °C to 900 °C, while the TG curve showed weight loss in two stages (red line). Initially, a broad endothermic peak appeared at 132 °C, corresponding to a 12.1 % weight loss, attributed to dehydration of S-CPP1 (Stage 1). Subsequently, a significant weight loss of 25.6 % aligned with two exothermic peaks at 305 °C and 490 °C, indicated the decomposition of organic components (primarily proteins) within S-CPP1 (Stage 2). Finally, another exothermic peak appeared at 688 °C, indicating the crystallization of S-CPP1 (Stage 3).

Compared to S-CPP1, L-CPP1 consisted of ellipsoidal nanoparticles with a relatively larger particle size of 70.6 ± 16.7 nm (Fig. 2b1,b2). In contrast, Fig. 2c1 shows that CPP2 comprised aggregated structures formed by HAP whiskers, with a particle size of 204 ± 41.3 nm (Fig. 2c2). The average hydrated diameters of L-CPP1 and CPP2, as determined by a particle size analyzer, were 150.1 nm and 254.2 nm, respectively (Fig. 2b3,c3), both larger than their actual particle sizes. TG-DTA analysis (Fig. 2b4,c4) revealed that during heating from 30 °C to 900 °C, L-CPP1 and CPP2 exhibited three stages similar to those observed in S-CPP1. The results indicated that the protein content of synthesized L-CPP1 was approximately 18.9 %, lower than that of S-CPP1 (∼25.6 %), while CPP2 had the lowest protein content ∼7.6 %. This reduction corresponds to the gradual transition from S-CPP1 to L-CPP1 and eventually to CPP2, during which the increasing deposition of calcium phosphate mineral became predominant.

The XRD patterns of synthetic S-CPP1, L-CPP1 and CPP2 were shown in Fig. 2d, further highlighting their structural difference. S-CPP1 and L-CPP1 exhibited a broad diffraction peak at approximately 2*θ* = 30°, indicating the characteristic of amorphous status. In contrast, CPP2 exhibited diffraction peaks at 2*θ* angles of 25.9°, 31.8°, 32.2°, and 32.9°, corresponding well to the (002), (211), (112), and (300) crystal planes of HAP, respectively (JCPDF#09–0432). Notably, the retention of amorphous features suggests that CPP2 exhibits partial crystallization or possesses low crystallinity.

To verify the presence of protein components in synthetic S-CPP1, L-CPP1 and CPP2, FTIR analysis was conducted (Fig. 2e). The characteristic absorption peak at 1036 cm^−1^ corresponded to the stretching vibration of PO_4_^3−^, while the peak at 567 cm^−1^ was associated with its bending vibration [29,30], consistent with the structural feature of HAP identified in the XRD pattern. Notably, a distinctive absorption peak near 1654 cm^−1^ was observed in the protein amide I region, which is attributed to the C=O stretching vibration of both the α-helix secondary structure and amino acids [31]. Furthermore, the peaks at 1521 cm^−1^ were due to the C-N stretching linked to carbonyl groups in the protein amide II region, whereas the peak at 1424 cm^−1^ corresponded to the N-H and O-H in-plane bending vibrations in the amide III region [31]. These findings indicate the incorporation of protein components into the CPPs structure.

The synthesis of CPPs was necessitated by the limited quantity of endogenous CPPs extracted from the 5/6 nephrectomy rat model. Results indicated that the composition and particle sizes of CPPs synthesized using a DMEM-based system closely resembled those of endogenous CPPs. Specifically, the sizes of synthetic S-CPP1, L-CPP1, and CPP2 were 39.2 ± 5.5 nm, 70.6 ± 16.7 nm, and 204 ± 41.3 nm, corresponding to endogenous S-CPP1 (<50 nm), L-CPP1 (50–100 nm), and CPP2 (>100 nm), respectively. Synthetic S-CPP1 and L-CPP1, composed of calcium phosphate and protein, exhibited amorphous characteristics, while CPP2 showed crystalline features similar to HAP, consistent with endogenous CPPs. Thus, synthetic CPPs can be effectively used in subsequent experiments instead of endogenous CPPs in blood vessels, as described in Section 3.1, providing a scientific basis for further investigation.

### 3.3 The interactions between CPPs and ECs

**Fig. 3:**
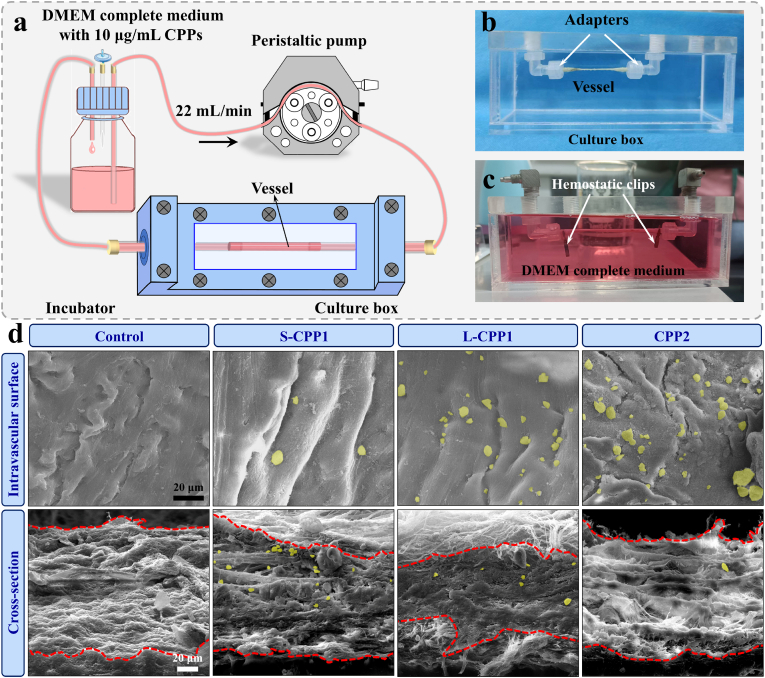
(a) Schematic diagram of *ex vivo* blood vessel model. (b) Photograph of the assembled vascular culture box. (c) Photograph of the culture box after the addition of the culture medium. (d) SEM micrograph of the intravascular side and cross-section of the abdominal aorta after 8 days of circulation, with calcification-like particles (highlighted by yellow color based on the gray values of the images) adhering to the tissue.

To investigate the dynamic interaction between CPPs and blood vessels, an *ex vivo* blood vessel model was constructed using rat abdominal aorta, as illustrated in the schematic diagram in Fig. 3a–c (details described in Section 2.3). The aorta was exposed to an 8-day circulation of different CPPs in a DMEM complete medium. As shown in Fig. 3d, compared to the control group, numerous particles (highlighted in yellow) were tightly adhered to the intravascular side and cross-section of the abdominal aorta in all experimental groups. EDS analysis of these particles (Fig. S3) confirmed the presence of Ca, P, C, and O elements, verifying the formation of calcification-like particles. Furthermore, when S-CPP1 nanoparticles was used in circulation, the number of calcification-like particles formed on the intravascular surface was the lowest, with L-CPP1 forming an intermediate quantity, and CPP2 producing the highest number of calcification-like particles. In contrast, when S-CPP1 was involved in circulation, the number of calcification-like particles in the vascular cross-section was the highest, with L-CPP1 forming an intermediate quantity and CPP2 producing the lowest number of calcification-like particles. H&E staining of the vessels showed that the vascular structure remained intact, with no damage observed in any of the CPPs-treated groups (Fig. S4). These findings infer that different CPPs induce the formation of varying numbers of calcification-like particles within the blood vessels, indicating the size-dependent interactions between CPPs and vascular endothelium.

Long-term mineral metabolism imbalance leads to the formation of CPPs in the blood of CKD patients. Michael et al. analyzed the serum of CKD peritoneal dialysis patients and found that the CPPs concentration was about 20–80 mg/mL [32]. This concentration is too low to conduct short-term *in vitro* experiments to study the interactions between CPPs and blood vessels. Therefore, higher concentrations of CPPs were used to accelerate the interactions between CPPs and HUVECs.

**Fig. 4:**
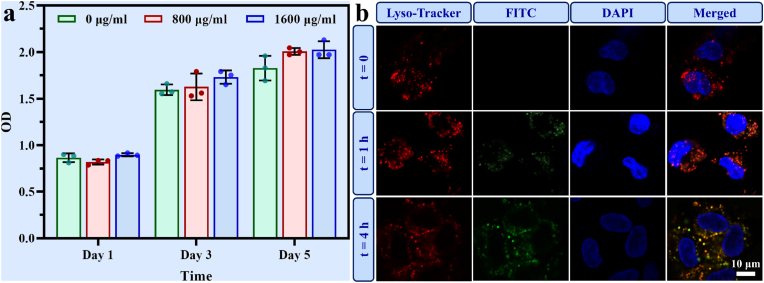
(a) OD values from the CCK-8 assay for HUVECs co-cultured with L-CPP1 at different concentrations for 1, 3, and 5 days. (b) CLSM images showing the internalization and co-localization of FITC-L-CPP1 with lysosomes in HUVECs. Cells were incubated with FITC-L-CPP1 for 0, 1 and 4 h. The Lyso-Tracker stain (red) marks the lysosomes, FITC (green) labels the internalized L-CPP1, and DAPI (blue) stains the cell nuclei. Scale bar: 10 μm.

The intravascular side consists of tightly packed ECs. To observe the internalization of L-CPP1 by these cells, its cytotoxicity was assessed. Specifically, HUVECs were co-cultured with L-CPP1 at different concentrations (0, 800, 1600 μg/mL) for 1, 3, and 5 days. The OD values from the CCK-8 assay steadily increased, indicating that co-culture with L-CPP1 did not affect cell proliferation under these conditions (Fig. 4a). To visualize the internalization of L-CPP1 by ECs, synthetic L-CPP1 was conjugated with fluorescein isothiocyanate (FITC-L-CPP1). The uptake and intracellular transport of FITC-L-CPP1 were monitored at different time points (1 and 4 h) using CLSM. At starting (t = 0), no green fluorescence was observed within the cells (Fig. 4b), indicating that no nanoparticle uptake had occurred. After 1 h of incubation (t = 1 h), an increase in green fluorescence intensity was detected inside the cells, signifying the continuous internalization of L-CPP1 by HUVECs over time. Furthermore, the obvious co-localization of FITC-L-CPP1 (green) with lysosomes (red), along with the cell nuclei (blue), confirmed that L-CPP1 was internalized through endocytosis by ECs.

**Fig. 5:**
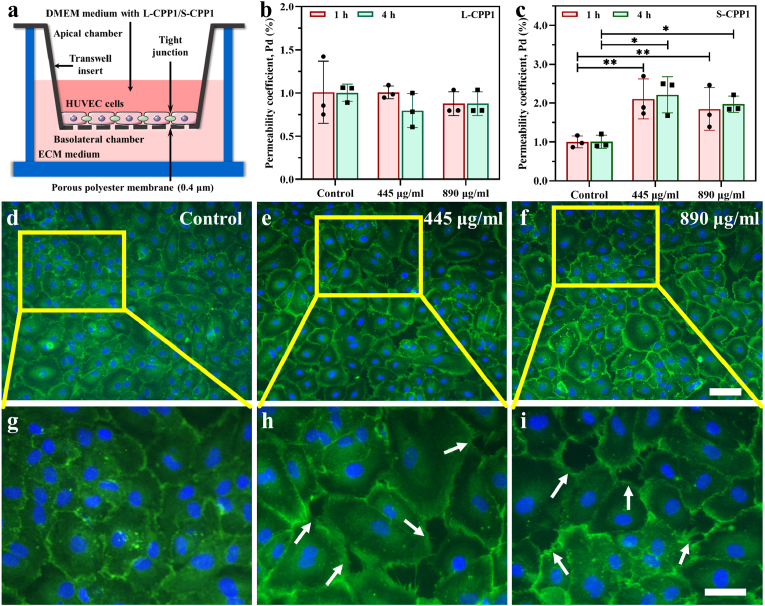
Assessment of endothelial cell permeability and integrity following exposure to L-CPP1 and S-CPP1. (a) Schematic diagram of the *in vitro* transwell system. (b) Permeability coefficients of HUVECs treated with different concentrations of L-CPP1 (445 μg/mL and 890 μg/mL) for 1 h and 4 h. (c) Permeability coefficients of HUVECs treated with S-CPP1 (445 μg/mL and 890 μg/mL) for 1 h and 4 h. Data represent the mean ± standard deviation (n = 3). One-way ANOVA followed by Tukey's post hoc test was used for statistical analysis. ∗*p* < 0.05, ∗∗*p* < 0.01. (d–f) Distribution of VE-cadherin in HUVECs exposed to different concentrations of S-CPP1. VE-cadherin was labeled by immunofluorescence (green), and cell nuclei were stained with DAPI (blue). Scale bar: 100 μm. (g–i) Enlarged images from the yellow rectangular boxes in (d–f), with white arrows indicating widened intercellular gaps and irregular cell borders. Scale bar: 50 μm.

To evaluate whether CPPs with different sizes can undergo the paracellular pathway to bypass the endothelial barrier, a transwell system was designed and constructed to monitor the permeability of the HUVECs following exposure to varying concentrations of L-CPP1 and S-CPP1 (Fig. 5a). To accelerate the *in vitro* experiments, ECM complete medium containing CPPs at concentrations of 450 μg/mL and 890 μg/mL were used for the experimental groups, which are approximately five and ten times of the CPPs concentration found in the serum of CKD peritoneal dialysis patients, respectively [32]. After incubation with HUVECs for an h, the permeability coefficients of L-CPP1 at concentrations of 445 μg/mL and 890 μg/mL were 1.00 % ± 0.07 % and 0.88 % ± 0.14 %, respectively, showing no significant difference from the control group (1.00 % ± 0.36 %) (Fig. 5b). After 4 h of incubation, the permeability coefficients were 0.79 % ± 0.20 % and 0.92 % ± 0.15 %, respectively, also indicating no significant difference from the control group (1.00 % ± 0.10 %). These results suggest that L-CPP1 particles do not interrupt the integrity of HUVECs. However, the permeability coefficients of S-CPP1 at concentrations of 445 μg/mL and 890 μg/mL were 2.10 % ± 0.51 % and 1.85 % ± 0.55 %, respectively, which were approximately twice that of the control group (1.00 % ± 0.15 %) after incubation with HUVECs for an hour. After 4 h of incubation, the coefficients increased to 2.21 % ± 0.46 % and 1.97 % ± 0.21 %, respectively, indicating the ECs leakage increased over time with exposure to S-CPP1.

To further investigate the mechanism by which S-CPP1 induces leakage in the dense ECs layer, we performed immunofluorescence staining on HUVECs. Fig. 5d and g shows that in the absence of S-CPP1, VE-cadherin was normally expressed, forming a ring-like pattern around the cells with a tightly packed arrangement. In contrast, after co-culture with S-CPP1 (Fig. 5e, f, h, i), the morphology of the cellular edges became irregular, and the endothelial gaps widened significantly, as indicated by the white arrows. The disruption of VE-cadherin's homophilic interactions likely contributed to the increased permeability and leakage observed in HUVECs exposed to S-CPP1.

This section firstly investigates the dynamic interactions between CPPs and blood vessels using an *ex vivo* rat abdominal aorta model. Over 8 days of exposure to varying concentrations of CPPs, calcification-like particles formed on the aorta. The number of particles on the vascular surface and cross-section varied depending on the CPP type, with the smaller S-CPP1 inducing the fewest particles on the surface but the most in the cross-section, while the larger CPP2 exhibited the opposite pattern. The section further explored the internalization of CPPs by the endothelial barrier. Confocal microscopy revealed that L-CPP1 was internalized by HUVECs via endocytosis, co-localizing with lysosomes. In contrast, S-CPP1 increased endothelial cell leakage compared to L-CPP1, likely due to its smaller size. Immunofluorescence staining further showed that S-CPP1 disrupted VE-cadherin at cellular junctions, contributing to increased permeability. These findings suggest that the size of CPPs plays a critical role in modulating endothelial barrier integrity.

### 3.4 The interactions between S-CPP1 and SMCs

**Fig. 6:**
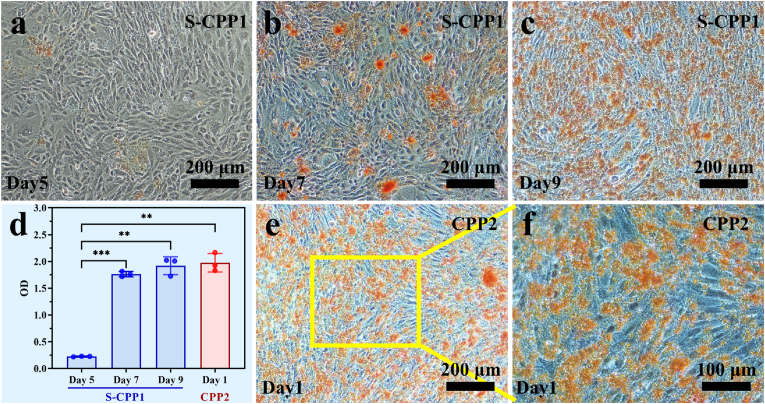
Evaluation of calcification in SMCs induced by S-CPP1 and CPP2. (a–c) Alizarin red S staining of A7r5 cells co-culture with 445 μg/mL S-CPP1 for 5, 7 and 9 days, respectively. Scale bar: 200 μm. (d) OD values for the quantitative analysis of Alizarin Red S staining of A7r5 cells under different experimental conditions. Data represent the mean ± standard deviation (n = 3). One-way ANOVA followed by Tukey's post hoc test was used for statistical analysis. ∗*p* < 0.05, ∗∗*p* < 0.01. (e, f) Alizarin Red S staining of A7r5 cells co-cultured with 200 μg/mL CPP2 for one day. Scale bar: 100 μm.

Blood vessels consist of three concentric layers: the tunica intima, media, and adventitia, which contain ECs, SMCs, and fibroblasts, respectively, all embedded within a highly organized extracellular matrix composed of collagen types I and III, and elastin. As demonstrated in Section 3.3, only S-CPP1 (<50 nm) can induce ECs leakage, allowing entry into the vessel interior, whereas L-CPP1 (50–100 nm) cannot disrupt EC junctions. Given that SMCs are the primary cell type involved in medial calcification [26], Alizarin Red S staining was used to evaluate the calcification of SMCs induced by S-CPP1. Fig. 6a–c showed the progressive formation of calcium nodules (orange) after co-culture of SMCs with 445 μg/mL S-CPP1 for 5, 7 and 9 days, respectively. The number of calcium nodules increased on day 7 and further escalated by day 9 (Fig. 6d). In contrast, extensive calcification was observed when SMCs were co-cultured with 200 μg/mL CPP2 for one day, as the positive control (Fig. 6e and f). The quantitative analysis illustrated that the calcium content in SMCs cultured with S-CPP1 for 9 days was comparable to that induced in SMCs cultured with CPP2 for only one day (Fig. 6d).

## 4 Discussion

Nanoparticles, with their unique physicochemical properties, have garnered significant attention in the medical field due to their potential for bioimaging, drug delivery, and other therapeutic treatments [2,33,34]. In nanoparticle-based drug therapy, intravenous injection causes nanoparticles to undergo transport through the bloodstream, cross the vascular endothelial barrier, and eventually reach the target site for drug delivery and absorption [3,35]. Therefore, understanding the interaction between nanoparticles and vascular endothelium is crucial for determining the effectiveness of the entire treatment process.

The interactions between nanoparticles and cells are influenced by various factors, particularly particle size, elasticity and surface characteristics. The size of nanoparticles shows significant influence on their interactions with tissues and cells. It is well-established that particles smaller than 20 nm are rapidly filtered by the kidneys, while those larger than 200 nm are captured and cleared by the reticuloendothelial system [36,37]. Therefore, researchers typically focus on nanoparticle diameters ranging from 20 to 200 nm to examine vascular penetration and particle distribution characteristics [3]. Nanoparticles traversed the ECs barrier of vessel primarily via two mechanisms [35]: the transcellular pathway, which involves the endocytosis of nanoparticles by ECs [38], and the paracellular pathway, characterized by the disruption of intercellular junctions to bypass the endothelial barrier [39]. Wang et al. found that Au nanoparticles with an initial size of 30 nm can induce endothelial leakage both *in vitro* and *in vivo*, forming intercellular gaps ranging from nanometers to several micrometers in diameter [16]. Nowak et al. found that low-toxicity Carboxylated Polystyrene (PS) nanoparticles exhibited a significant size-dependent interaction with the endothelium [37]. The 200 nm PS particles demonstrated the highest endothelial permeability, with their transport rate approximately three times greater than that of the 100 nm particles. The permeability of 500 nm particles was significantly lower, with their transport rate nearly 100 times less than that of the 200 nm particles. In addition, the elasticity of nanoparticles influences their interactions with cells. A previous study indicated that most inorganic nanoparticles, such as SiO_2_, TiO_2_, and HA, exhibit high stiffness (elastic modulus >10 GPa), whereas organic nanoparticles, such as PEG, polyglycolic acid (PGA), and poly (lactic-coglycolicacid) (PLGA), usually exhibit low stiffness (elastic modulus <0.1 GPa) [40]. The inorganic-organic hybrid nanoparticles or composites (such as CPPs) exhibited medium stiffness (0.1 GPa < elastic modulus <10 GPa). Therefore, the interactions between the inorganic/organic nanoparticles and cells may be different from that between CPPs and cells due to the significant difference in particle elasticity. Moreover, surface properties of these inorganic nanoparticles (e.g. Au) and organic nanoparticles (e.g. PS) are very different from CPPs. Therefore, CPPs provide a unique model to study interactions between nanoparticles and ECs, as they represent endogenous particles that exist in biological systems rather than synthetic constructs. Considering CPPs with different particle sizes show similar elasticity and surface characteristics. Therefore, we focus on investigation of the size-dependent interactions between CPPs and vascular endothelium in this study.

Recent studies have shown that mineral metabolism dysregulation in the serum of CKD patients leads to the formation of CPPs of varying sizes [19,20], including primary CPPs (CPP1) composed of amorphous calcium phosphate, and secondary CPPs (CPP2), which contain crystalline forms, contributing to vascular medial calcification [23,24]. Based on these findings, the formation and development of endogenous CPPs in a 5/6 nephrectomy rat model were investigated in this study. The results indicated that three weeks post-surgery, oval-shaped S-CPP1 particles smaller than 50 nm were detected in the bloodstream. After five weeks, larger L-CPP1 particles formed (approximately 50–100 nm), with some particles showing early crystallization and transitioning towards CPP2. Concurrently, calcified particles ranging from 5 to 20 nm were observed in the rat aorta, with both their composition and size similar to those of S-CPP1. These findings suggest that endogenous CPPs of varying sizes can affect the vascular medial calcification. The CPPs observed in CKD patients will serve as a practical reference model for studying the interaction between nanoparticles and the vascular endothelium.

**Fig. 7:**
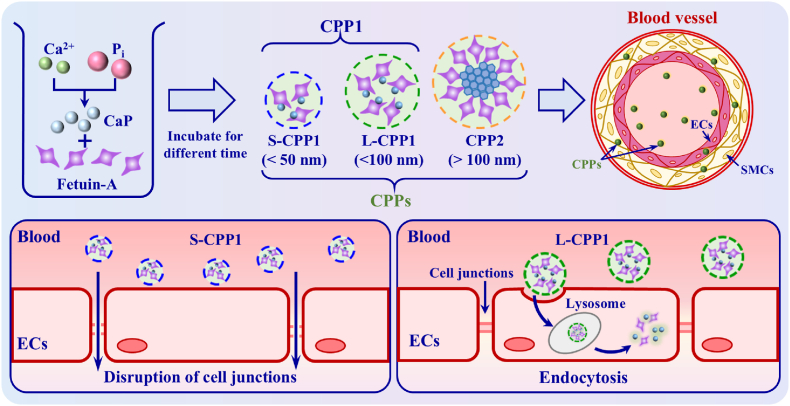
Schematic diagram illustrating the formation and maturation of CPPs and the formation of calcification-like particles in blood vessels. For CKD patients, elevated levels of Ca^2+^ and PO_4_^3−^ (Pi) contribute to the formation of CaPi ion clusters. These CaPi ion clusters are tightly bound and primarily stabilized by fetuin-A, forming the basic components of S-CPP1 (<50 nm), known as calciprotein monomers. Over time, S-CPP1 can grow and increase in size, forming L-CPP1 (50–100 nm). CPP1 can undergo further aggregation, transitioning from the amorphous phase to the crystalline phase of calcium phosphate, eventually forming large CPPs (>100 nm). S-CPP1 can disrupt endothelial cell junctions, allowing them to penetrate the vascular media composed of SMCs, while L-CPP1 is internalized via endocytosis by ECs.

The concentration of CPPs in the serum of CKD patients is extremely low (only 20–80 μg/ml) [32]. Additionally, vascular calcification in these patients is a slowly progressing pathological process, spanning months to years [41]. Therefore, using low physiological concentrations of CPPs in *ex vivo* or *in vitro* experiments could result in insufficient interaction with the vascular endothelium in a short time period (e.g. 1–2 weeks). On the other hand, *ex vivo* or *in vitro* experiments with physiological concentrations for the CKD model over extended periods (months or years) would result in significant challenges in terms of experimental complexity and precision, due to the aggregation of CPPs in the medium. Therefore, the concentrations of CPPs used for *ex vivo* and *in vitro* experiments were amplified to accelerate the interaction between CPPs and ECs in this study. Our findings demonstrated that S-CPP1 induced significant leakage in HUVECs within an hour, likely attributed to its small particle size (<50 nm) that can disrupt intercellular VE-cadherin interactions of ECs. The extent of leakage increased notably after 4 h. In contrast, L-CPP1, due to its larger particle size mainly ∼50–100 nm, were mostly internalized by ECs through endocytosis. Once inside the cells, the internalization of L-CPP1 raised intracellular Ca^2+^ levels, leading to the inflammatory activation of ECs. A previous study also discovered that CPPs endocytosed by HK-2 epithelial cells could lead to lysosomal swelling and nanoparticle accumulation within lysosomes, ultimately resulting in lysosomal dysfunction [42]. ECs serve as a barrier between CPPs and the underlying vascular tissue, representing the vascular tissue directly exposed to CPPs. CPPs can activate endothelial inflammation through the nuclear factor-κB (NF-κB) pathway, leading to endothelial dysfunction and promoting vascular calcification [43]. Additionally, *in vitro* experiments have shown that when CPPs interact with SMCs, the elevated intracellular Ca^2+^ and PO_4_^3−^ concentrations can trigger osteochondrogenic dedifferentiation, a process that leads to the production and release of calcifying microvesicles, thereby promoting vascular calcification [44]. Furthermore, ECs may indirectly promote SMCs calcification by increasing the release of pro-calcification molecules (e.g., IL-6) or extracellular vesicles [45]. However, whether this occurs *in vivo* requires further experimental validation. As the levels of CPPs increase in the blood of CKD patients, vessel calcification continues to accumulate, ultimately exacerbating calcification (Fig. 7).

The pathophysiological mechanisms of vascular calcification in CKD patients are complex, involving processes such as CPPs internalization, inflammatory signaling, and oxidative stress. Understanding how CPPs of different sizes interact with and cross the vascular endothelium holds significant clinical relevance. The endothelium acts as a critical barrier that regulates the movement of molecules and particles between the bloodstream and surrounding tissues. By investigating the size-dependent permeability of CPPs, we can better understand how CPPs may influence vascular health, particularly in conditions such as inflammation, atherosclerosis, and vascular calcification. This knowledge could lead to the development of targeted therapeutic strategies that harness nanoparticle characteristics to improve drug delivery or mitigate endothelial dysfunction. Additionally, this research could provide insights into the mechanisms by which various nanoparticles, including those used in drug delivery systems, interact with the endothelium, thus aiding the design of safe and more effective treatments.

## 5 Conclusions

Understanding how calciprotein particles (CPPs) of different sizes interact with and cross the vascular endothelium, a crucial barrier regulating molecular and particle movement, is of significant clinical importance. In this study, we used a chronic kidney disease (CKD) rat model induced by 5/6 nephrectomy as an *in vivo* system to investigate the interactions between CPPs and blood vessels. The results demonstrated that small-sized CPPs (S-CPP1, <50 nm) appeared in the blood after three weeks, while larger CPPs (L-CPP1, 50–100 nm) formed after five weeks. We successfully synthesized three types of CPPs: ellipsoidal S-CPP1 (<50 nm), L-CPP1 (50–100 nm), and secondary CPPs (CPP2, >100 nm), characterized by needle-like crystalline HAP. Our findings showed that L-CPP1 is internalized by ECs through endocytosis without affecting endothelial permeability. In contrast, S-CPP1 disrupted VE-cadherin junctions, leading to altered cell morphology and widened endothelial gaps. This disruption allowed S-CPP1 to penetrate the vascular media, where it induced calcification in SMCs. Overall, this study provides valuable insights into the size-dependent interactions between nanoparticles and blood vessels, advancing our understanding of vascular calcification and highlighting potential implications for nanoparticle-based therapies in CKD. The ability of nanoparticles to traverse the vascular endothelium, particularly in a size-dependent manner, offers promising potential for targeted drug delivery and advancing therapies.

## CRediT authorship contribution statement

**Zeping Zhang:** Writing – review & editing, Writing – original draft, Formal analysis. **Xinyue Wang:** Writing – original draft, Methodology, Investigation, Formal analysis, Data curation. **Caihao Huang:** Writing – review & editing, Data curation. **Meixia Wang:** Methodology, Investigation, Formal analysis, Data curation. **Wei Cui:** Methodology, Investigation, Formal analysis, Data curation. **Liang Hao:** Investigation, Funding acquisition, Formal analysis. **Rui Yang:** Writing – review & editing, Supervision, Investigation. **Hong-hui Wang:** Writing – review & editing, Writing – original draft, Supervision. **Xing Zhang:** Writing – review & editing, Writing – original draft, Supervision, Funding acquisition, Conceptualization.

## Declaration of competing interest

The authors declare that they have no known competing financial interests or personal relationships that could have appeared to influence the work reported in this paper.

## Data Availability

Data will be made available on request.
