# Supplementary material for: Size-dependent interactions between calciprotein particles and vascular endothelium
Source: Mater Today Bio. 2025 Feb 19;31:101599. doi: 10.1016/j.mtbio.2025.101599 (PMC11894339; doi:10.1016/j.mtbio.2025.101599)
Supplement: Multimedia component 1 [file mmc1.docx]

Supporting Information for

**Size-dependent Interactions between Calciprotein Particles**

**and Vascular Endothelium**

Zeping Zhang^a,b,1^, Xinyue Wang^a,b,1^, Caihao Huang^a,c^, Meixia Wang^d^, Wei Cui^a,b^, Liang Hao^e^, Rui Yang^a,b^, Hong-hui Wang^d,*^, Xing Zhang^a,b,*^

^a^ *Institute of Metal Research, Chinese Academy of Sciences, Shenyang, Liaoning 110016, China*

^b^ *School of Materials Science and Engineering, University of Science and Technology of China, Hefei, Anhui 230026, China*

^c^ *School of Materials Science and Engineering, Dalian University of Technology, Dalian, Liaoning 116024, China*

^d^ *State Key Laboratory of Chemo/Bio-Sensing and Chemometrics, College of Biology, Hunan University, Changsha, Hunan 410082, China*

^e^ *School of Forensic Medicine, China Medical University, Shenyang, Liaoning 110026, China*

^*^ Corresponding authors:

*E-mail address:* *E-mail address:* [wanghonghui@hnu.edu.cn](mailto:wanghonghui@hnu.edu.cn) (H. Wang); [xingzhang@imr.ac.cn](mailto:xingzhang@imr.ac.cn) (X. Zhang).

^1^ These authors contributed equally to this work.


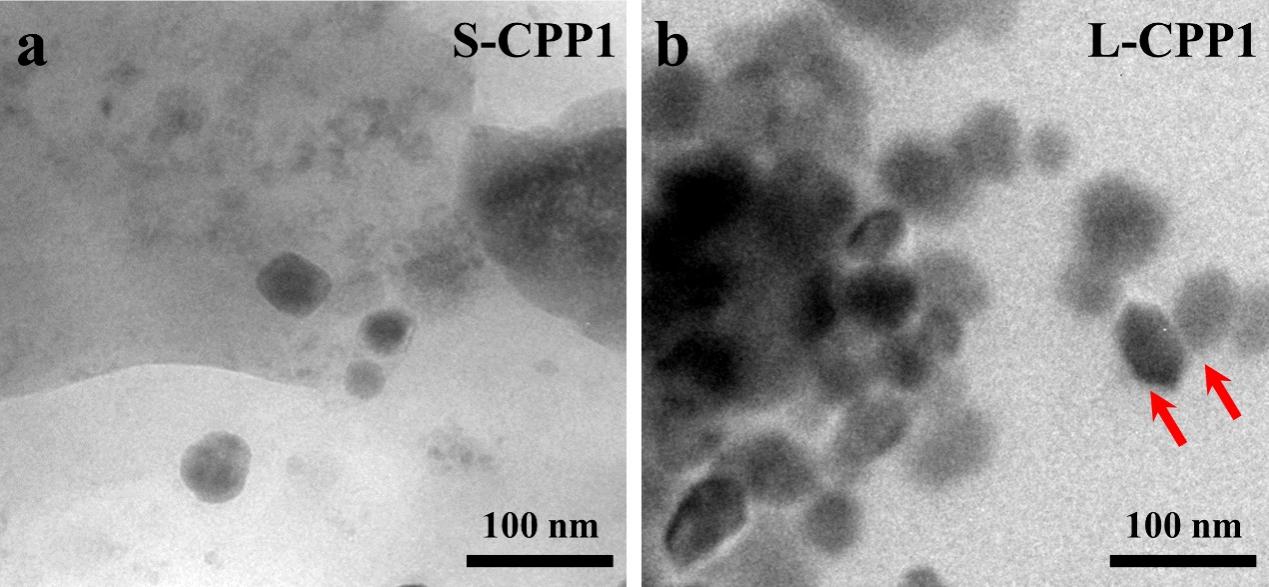


Fig. S1. High-resolution TEM images of endogenous S-CPP1 and partially crystallized L-CPP1 isolated from blood at (a) 3 weeks and (b) 5 weeks following the construction of the 5/6 nephrectomy rat model.


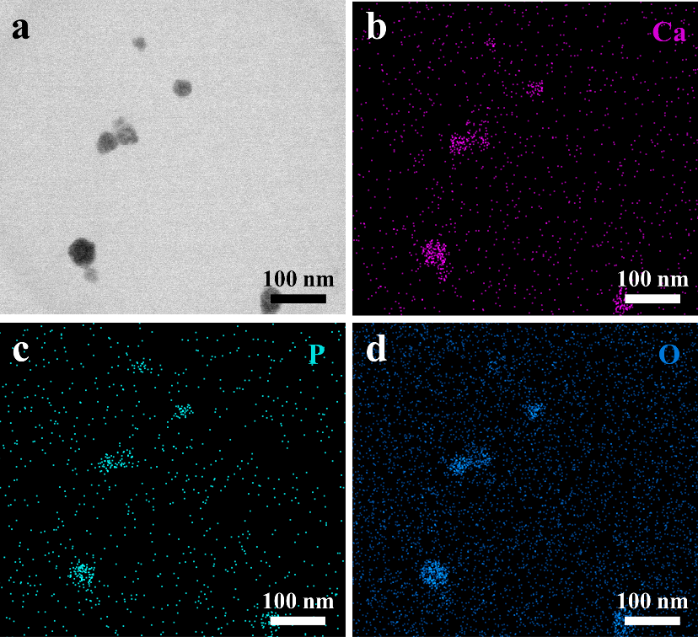


Fig. S2. (a) TEM bright field image of S-CPP1 incubated for 4 h and (b-d) the corresponding element mapping of Ca, P and O characterized by EDS.


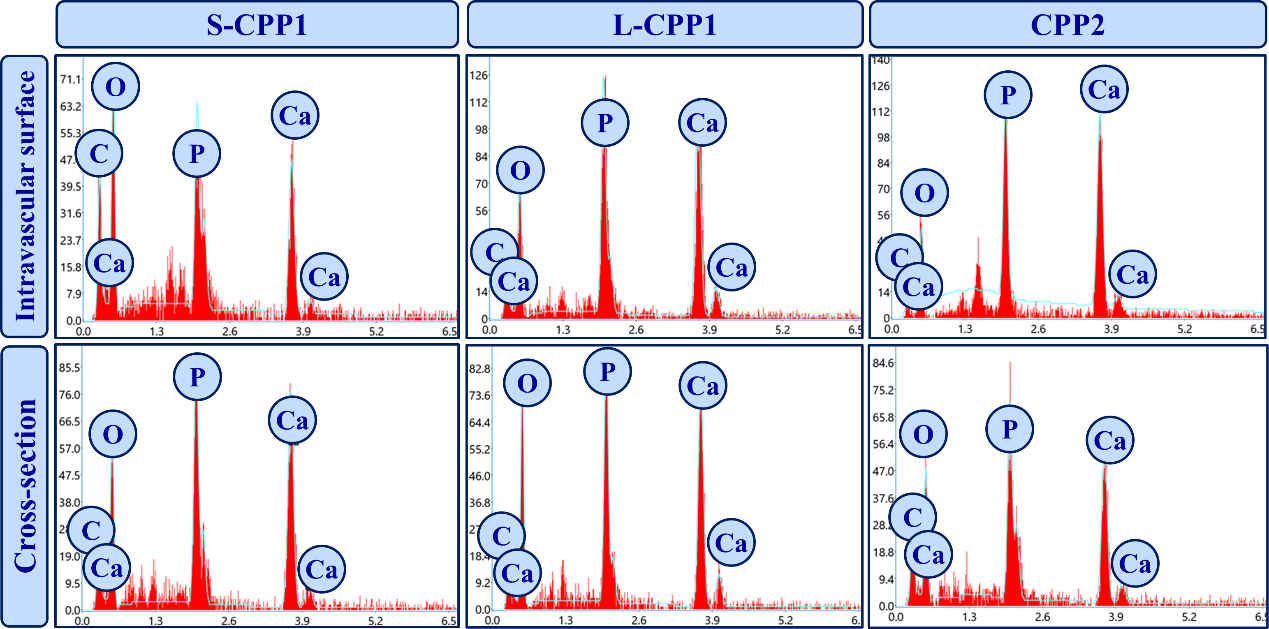


Fig. S3. EDS analysis of the particles formed on the intravascular side and cross-section of the rat abdominal aorta *in vitro* vascular model after treatment with different CPPs.


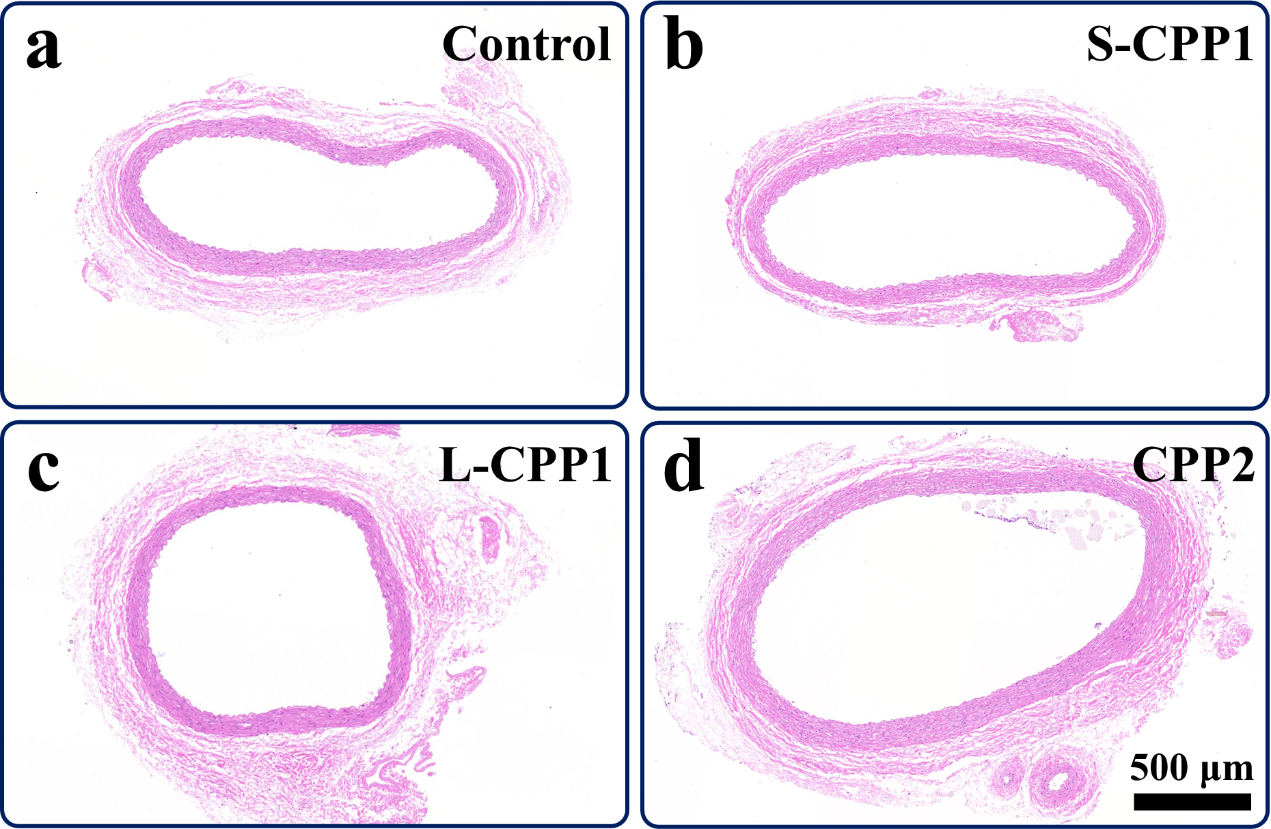


Fig. S4. H&E stained images of the rat abdominal aorta *in vitro* vascular model after treatment with different CPPs
